# Cellulose-Based Nanofibers Processing Techniques and Methods Based on Bottom-Up Approach—A Review

**DOI:** 10.3390/polym14020286

**Published:** 2022-01-11

**Authors:** Ana Kramar, Francisco Javier González-Benito

**Affiliations:** Department of Materials Science and Engineering and Chemical engineering, Instituto Tecnológico de Química y Materiales “Álvaro Alonso Barba”, Universidad Carlos III de Madrid, Avda. Universidad 30, 28911 Leganés, Spain; javid@ing.uc3m.es

**Keywords:** cellulose, nanofibers, cellulose derivatives, spinning techniques

## Abstract

In the past decades, cellulose (one of the most important natural polymers), in the form of nanofibers, has received special attention. The nanofibrous morphology may provide exceptional properties to materials due to the high aspect ratio and dimensions in the nanometer range of the nanofibers. The first feature may lead to important consequences in mechanical behavior if there exists a particular orientation of fibers. On the other hand, nano-sizes provide a high surface-to-volume ratio, which can have important consequences on many properties, such as the wettability. There are two basic approaches for cellulose nanofibers preparation. The top-down approach implies the isolation/extraction of cellulose nanofibrils (CNFs) and nanocrystals (CNCs) from a variety of natural resources, whereby dimensions of isolates are limited by the source of cellulose and extraction procedures. The bottom-up approach can be considered in this context as the production of nanofibers using various spinning techniques, resulting in nonwoven mats or filaments. During the spinning, depending on the method and processing conditions, good control of the resulting nanofibers dimensions and, consequently, the properties of the produced materials, is possible. Pulp, cotton, and already isolated CNFs/CNCs may be used as precursors for spinning, alongside cellulose derivatives, namely esters and ethers. This review focuses on various spinning techniques to produce submicrometric fibers comprised of cellulose and cellulose derivatives. The spinning of cellulose requires the preparation of spinning solutions; therefore, an overview of various solvents is presented showing their influence on spinnability and resulting properties of nanofibers. In addition, it is shown how bottom-up spinning techniques can be used for recycling cellulose waste into new materials with added value. The application of produced cellulose fibers in various fields is also highlighted, ranging from drug delivery systems, high-strength nonwovens and filaments, filtration membranes, to biomedical scaffolds.

## 1. Introduction

Cellulose is the most abundant natural polymer on Earth [1]. The ambiguous properties of cellulose make this polymer the most studied in terms of its production and processing. On the one hand, cellulose is renewable, non-toxic, and biodegradable; on the other hand, it is highly crystalline, degrades before melting (and is therefore not suitable for melt-spinning-based techniques), and is very hard to dissolve in common solvents, although it is very hydrophilic because of an abundance of hydroxyl groups. Therefore, special attention must be paid to identifying appropriate techniques to produce cellulose nanofibers from solutions and, thus, the conditions under which they should work to control the final morphology.

The emergence of polymeric nanofibers spinning dates back to the late 20th century, with techniques adapted for thermoplastic polymers. The most utilized and famous technique, which is widely used for polymer nanofibers preparation, is electrospinning [2,3,4]. Other techniques include hybrid melt electrospinning [5], melt spinning [6], bicomponent fiber spinning, drawing, and centrifugal spinning [7]. The application of nanofibers in general ranges from advanced food packaging systems [8,9,10], drug delivery and tissue engineering [2,11,12,13], to filtering membranes for water [4,14] and air pollutants [15]. Nanofibrous membranes, besides their microporosity, which comes from the structure of membrane itself containing entangled nanofibers, possess nanoporosity originating from porous nanofibers. The overall permeability of nanofibrous membranes is therefore important [16], since it enables a range of applications, including in fuel cells, battery separators, supercapacitors, protective textile, etc. The great variety of possible applications has caused increased interest in exploring and establishing more methods and techniques for nanofibers production as well as new polymer-based systems to be used as a precursor for such production.

Historically, the first-ever man-made fiber of macroscopic diameter in the beginning of the 20th century was regenerated cellulose fiber, so-called viscose rayon, using the conventional wet spinning technique [1,17]. Interestingly, the second man-made fibers were also cellulose-based fibers, made from an ester of cellulose, acetate, produced industrially long before other thermoplastic fibers. So, naturally, with the emergence of nanofibers, the interest in the production of cellulose-based nanofibers is expected. This review is focused on the latest achievements and potential future lines in the field of spinning techniques for cellulose nanofiber production. Two distinctive approaches are currently utilized when dealing with cellulose nanofibers, the so-called top-down approach in which cellulose nanofibers and nanocrystals are isolated from various cellulose sources, and the one, bottom-up approach, wherein cellulose nanofibers are spun from precursors into non-aligned or aligned filaments to generate mats or membranes [18].

Many review articles are focused on the extraction and purification of CNFs or CNCs using a top-down approach. However, in this review, those are not included; instead, CNFs are mentioned as a precursor for spinning. Readers are encouraged to read high-quality reviews regarding top-down approaches for cellulose nanofibrils and nanocrystals isolation and preparation [19,20,21]. Moreover, scientific works in which CNFs or CNCs were used only as reinforcement for other polymers (not as composite) have also been excluded from this review, except when they were used with cellulose or its derivatives to prepare cellulose-based composites.

**Figure 1:**
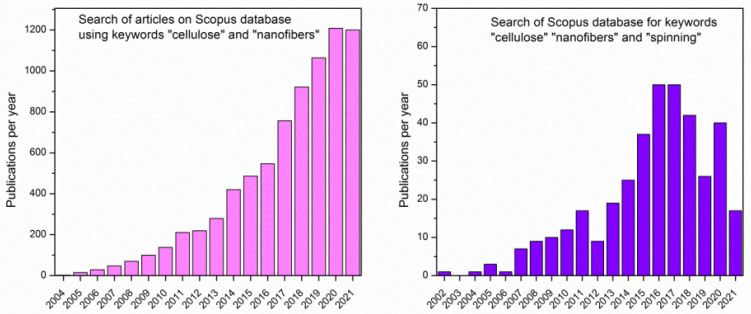
Number of publications per year from database Scopus using keywords “cellulose” and “nanofibers” (**left**) and refined search including the word “spinning” (**right**).

The publication’s analysis has been focused on using the database Scopus up to September 2021, using the keywords “cellulose” and “nanofibers” (Figure 1). The search led to over 7400 results in the form of different scientific publications for the period of 2000 to 2021. Additional refining of the results has been performed using the word “spinning”, which led to over 700 articles. After careful reading of articles, publications wherein cellulose fibers were used as reinforcement of other polymeric materials and those including bacterial cellulose have been excluded. The resulting distribution of publications per year (Figure 1) indicates an increasing trend in research interest in the spinning of cellulose fibers, of which almost 50% is focused on the very popular electrospinning technique.

Electrospinning, ES, is a technique that was utilized for the first time to process cellulose-based derivative, cellulose acetate, in 2002 [22], followed by other publications in 2005 where ES was used for the preparation of pure cellulose fibers [23,24]. The next several years marked the intensive growth of research articles where various groups all over the world started to investigate different possible techniques for cellulose nanofiber production so that, with the advent of new spinning technologies, new horizons in terms of novel applications have been possible. As an alternative to electrospinning, there is an emerging technique, solution blow spinning (SBS), which is showing great potential for cellulose nanofibers preparation [25] and will be discussed in this review.

Currently, a high number of studies have presented the latest achievements, mostly in the electrospinning technique, for cellulose nanofibers production [26,27,28,29] or techniques for aligning the isolated CNCs or CNFs into more complex structures [18,30]. However, a comprehensive review of all the currently used techniques for cellulose nanofibers processing has not been presented. For the future directions of research and to identify potential gaps in the literature, it is important to summarize the currently available techniques for cellulose nanofibers preparation from various resources.

Since cellulose has limitations regarding its solubility in various solvents and the impossibility of being melted, the production or spinning of cellulose nanofibers is very challenging. However, in recent years, many researchers have been breaking barriers and pushing the technology further to find out ways to first dissolve the cellulose and then successfully spin the nanofibers from a solution. One of the breakthroughs is the use of ionic liquids [26,31], which enable fast and complete dissolution of cellulose, furthermore, they have been also used for the preparation of spinning dopes. To properly perceive all factors affecting the processing of cellulose to produce nanofibers, it is important at first to make a short overview of its structure and related properties especially solubility since the preparation of cellulose solutions is a crucial previous step within the whole spinning process.

The main objective of this study is to identify and present major techniques for cellulose nanofibers production, based on the source of cellulose (pulp, CNCs or CNFs, cellulose derivatives), parameters of the used method, especially solvent for the preparation of spinning dope, and the potential application of prepared nanofibrous materials.

## 2. Cellulose, Structure, Solubility and Cellulose Derivatives

**Figure 2:**
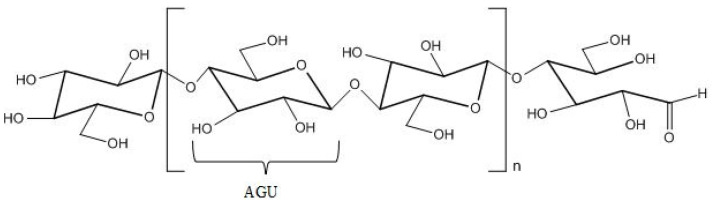
Chemical structure of cellulose.

Cellulose is a linear polymer comprised of D-glucopyranose units linked via β-1-4 glycoside bond [1,32]. It is a stereoregular, syndiotactic polymer with highly ordered molecular and supramolecular structure. The macromolecule of cellulose has four hydroxyl (OH) groups on one end, while on the other end, it has a carbonyl (aldehyde) group, which is frequently in the hemiacetal form, and it is the only carbonyl present in the cellulose [33]. Even though it has three hydroxyl groups on each anhydroglucose unit (AGU) (Figure 2), cellulose is not soluble in water, because these groups are involved in intra- and inter-molecular hydrogen bonds, leading to a complex and highly ordered network with a high degree of crystallinity. In fact, this ordered structure causes many common organic solvents to be unable to dissolve cellulose, both totally and partially. The physico-chemical properties of cellulose are highly dependent on the source of cellulose. Table 1 provides some important physico-chemical properties of cellulose based on the source of cellulose [1,17,32].

Due to the high number of hydroxyl groups, cellulose can also be transformed in other derivatives from common reactions such as esterification, producing esters, the most important being cellulose acetate (acetyl cellulose) and nitrocellulose.

Moreover, cellulose can be converted to corresponding ethers as well, by reactions of etherification, thus producing important derivatives such as alkyl celluloses and hydroxyalkyl celluloses.

Cellulose does not melt, but it degrades at high temperatures. Therefore, when processing cellulose, it is important to have a deep knowledge of its solubility as well as the properties of its derivatives since they might be completely different from those of the cellulose itself as a consequence of hydroxyl group substitution.

The solubility of cellulose is dependent both on the molecular weight and polydispersity [1]. Long molecular chains and high polydispersity together with the hydrogen bond network make cellulose very hard to dissolve. Few liquids are able to make cellulose swell but not dissolve completely; during swelling and partial dissolution, liquid penetrates the cellulose structure but large solid residue remains [1].

On the other hand, when dissolution of cellulose is considered, certain discrepancies appear in terms of supramolecular structure and dispersion of macromolecules. Liquid penetrates the cellulose structure, causing intermolecular hydrogen bonds to break and macromolecules to disperse. According to a specific interaction of cellulose with a particular solvent, the classification of cellulose solvents can be made on the basis of derivatizing and non-derivatizing solvents, as stated by Heinze and Koschella [34].

Non-derivatizing solvents are the ones that are involved only in intermolecular interaction, while derivatizing solvents or systems are those involved in a dissolution accompanied with the formation of unstable ether or ester derivatives. The covalent derivatization must be reversible, i.e., the formed derivative must be regenerated back to cellulose by a simple change of medium (e.g., from non-aqueous to aqueous) or just the pH [34]. Aqueous non-derivatizing solvents are solutions of inorganic salts and complex compounds such as cuprammonium hydroxide (Cuam) and cupriethylenediamine hydroxide (Cuen). The most famous and utilized non-aqueous non-derivatizing solvent is the system lithium chloride/*N*, *N*—Dimethylacetamide (LiCl/DMAc). It is frequently used in cellulose analytics [35] and is suitable for high-molecular-weight celluloses such as cotton or bacterial cellulose [34]. Another non-derivatizing solvent worth highlighting here is *N*-methylmorpholine-*N*-oxide (abbreviated as NMMO and NMNO), which is used in the production of Lyocell fibers. NMMO is a non-toxic recyclable solvent for cellulose [34,36]. Ionic liquids (ILs) is the term used for naming a group of organic salts which are liquid at a temperature lower than 100 °C, with low vapor pressure, and high chemical and thermal stability, and they are consider green, eco-friendly solvents [37]. IL anions form hydrogen bonds with cellulose and solubilize it through a non-derivatizing process [31] and by the simple addition of water or alcohol, cellulose is regenerated from the solution. The commonly used ILs for cellulose are 1-buthyl-3 methylimidazolium chloride BMIMCl and 1-allyl-3 methylimidazolium chloride (AMIMCl) [37]. As will be shown later, in this review, a successful use of ILs for the preparation of cellulose spinning dope has been reported.

Dimethyl sulfoxide/tetrabutylammonium fluoride trihydrate (DMSO/TBFA) is reported as another powerful non-derivatizing solvent for cellulose [34]. It is interesting to highlight here, as the authors pointed out, that dissolution does not occur if halide is changed; therefore, only fluoride can be used as a solvent [34]. As an example of derivatizing solvent, *N*,*N*-dimethylformamide/dinitrogen tetroxide (DMF/N_2_O_4_) and trifluoroacetic acid (TFA) are worth mentioning, whereby intermediate is formed during dissolution, namely cellulose nitrite and cellulose trifluoroacetate, respectively [34].

## 3. Spinning of Cellulose Nanofibers

**Figure 3:**
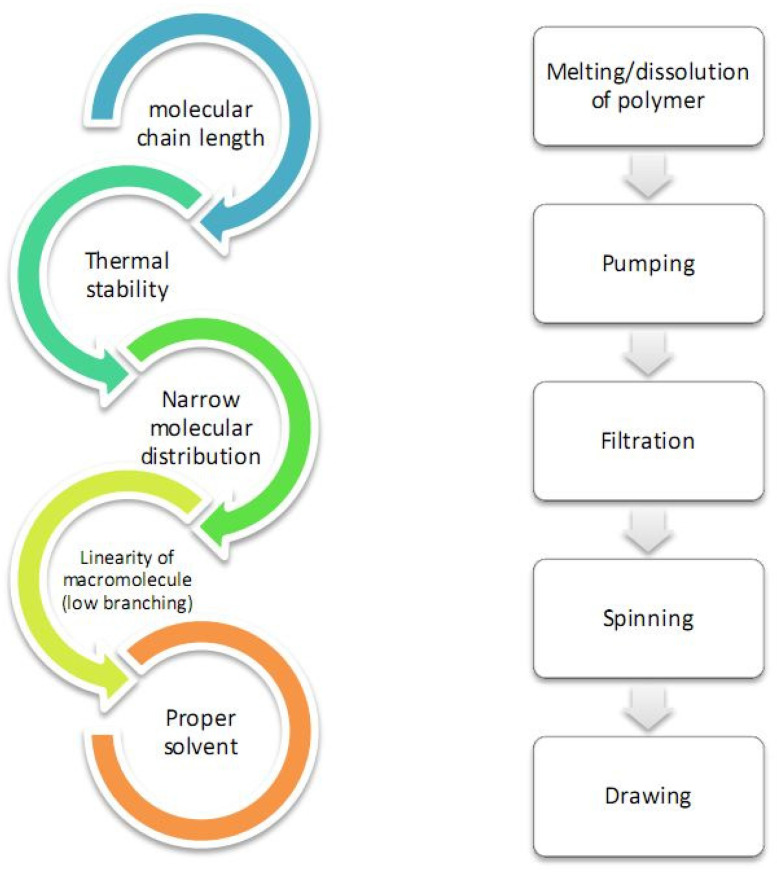
Some important properties of polymers for ensuring spinnability and simplified scheme of steps during spinning.

Spinning of polymeric fibers in general requires several steps to be performed, which depend mostly on properties of the polymer to be spun, for example, solubility and thermal behavior or whether the polymer can be melted without degradation. Some of the main characteristics that make the polymer spinnable are highlighted in Figure 3. General steps for polymer-based fiber spinning are also presented in Figure 3 in a simplified scheme. Since cellulose does not melt, its dissolution in a proper solvent is a crucial step. Therefore, in the nextsections, special attention will be given to the solvent used for preparation of spinning dopes because, as it will be shown, the properties of the corresponding solution greatly determine the techniques and processing conditions that must be used for spinning.

For spinning of cellulose nanofibers, several techniques are being used, most frequently electrospinning, dry spinning, solution blow spinning, wet spinning, hybrid dry-jet wet spinning and recently, an interfacial polyelectrolyte complex (IPC) spinning (Table 2).

Which technique will be utilized mostly depends on the precursor for spinning (source of cellulose) and type of solvent (whether or not it is volatile). The volatility of the solvent greatly influences the technique for fiber preparation. When the solvent is volatile, removal of it during the formation of fibers could be achieved by simple evaporation, either by air blowing, as in solution blow spinning, or collector heating, as in some hybrid setups for electrospinning. On the other hand, for a complete removal of non-volatile solvents, a coagulation bath is used, which is common for wet spinning techniques. As will be presented later in this review, there are several examples of hybrid techniques, which involve the additional heating of some part of the spinning device (the needle or collector) or adding a coagulation bath or water mist chambers in blow spinning techniques, to improve the removal of the solvent and formation of fibers.

As it is represented in Table 2, for electrospinning and solution blow spinning, cellulose from cotton and pulp is frequently used. On the other hand, CNFs are usually processed using dry spinning techniques, followed by wet spinning and the dry-jet wet spinning technique for the orientation of CNFs into more complex structures.

### 3.1. Electrospinning

Electrospinning is the most famous and utilized technique for nanofiber production. Some important requirements of the solvent for successful electrospinning of nanofibers from polymer solution, as stated by Ohkawa et al. [38], are: “(1) semi-conductivity with moderate charge capacity; (2) high volatility to facilitate solidification of polymer fibers; and (3) ability to dissolve the polysaccharides with less intermolecular interactions”. Having in mind the complexity of the cellulose structure and properties, as well as its solubility, there are still many challenges that need to be overcome for cellulose nanofibers to be produced using electrospinning.

However, successful preparation of pure cellulose nanofibers utilizing electrospinning have been reported by several authors in 2005 [23,24] from cellulose solution in NMMO. For instance, Kulpinski studied the optimization of cellulose nanofibers production using dry-wet electrospinning [23]. Firstly, cellulose from spruce pulp was mercerized with NaOH and afterwards dissolved in commercial 50% NMMO solution. The concentration of cellulose in NMMO varied between 1 and 4 wt%. Due to the fact that NMMO solvent of cellulose crystalizes upon cooling [60], spinning dope was heated and electrospinning was carried out at temperatures between 80 and 100 °C. The spinning nozzle was at a distance of 10–15 cm from the collector (working distance) and the collector was submerged in a coagulation bath containing water or water with surfactants during the spinning. Kulpinski stated that successfully spun fibers were collected when a solution of 2 wt% of cellulose was used, while a concentration of 1 wt% was too low for the formation of nanofibers. On the other hand, 4 wt% was too viscous to properly allow the process, leading to only droplets because the applied voltage was unable to induce the formation of a Taylor cone and, ultimately, the nanofibers. The fibers obtained from 2 wt% solution had an average diameter below 500 nm. Interestingly, when the surface of the coagulation bath was static, film-like material was produced, while the surface motion enabled the formation of fibers [23].

Khil et al. [24] took this a step further, processing cellulose by electrospinning followed by oxidation of cellulose nanofibers using NO_2_ in order to introduce carboxyl groups onto the polymer, thus making it applicable in the medical field. Namely, oxycellulose with a high content of carboxyl groups is biocompatible, biodegradable, and can be used to stop bleeding during surgery, or to prevent the formation of post-surgical adhesions. Here, the authors reported the preparation of cellulose nanofibers from 4 wt% solution of cellulose in 50% NMMO commercial solvent. Moreover, the authors used dry electrospinning, without a coagulation bath, and at room temperature. The obtained nanofibers had diameters between 90 nm and 250 nm, which were later subjected to oxidation, producing oxycellulose nanofibers. Khil and coworkers used a higher voltage (15 kV) compared to Kulpinski (9–9.5 kV), which is probably one of the reasons why Khil and coworkers were successful in obtaining nanofibers from more a viscous cellulose solution.

**Figure 4:**
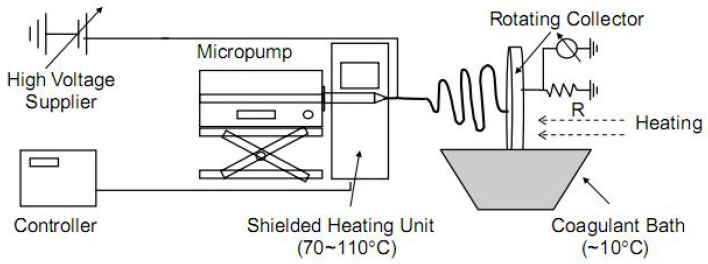
Scheme of the electrospinning system used for production of cellulose fibers (reprinted from *Polymer*. **2006**, *47*, 5097–5107, Kim, C.W.; Kim, D.S.; Kang, S.Y.; Marquez, M.; Joo, Y.L. Structural studies of electrospun cellulose nanofibers, with permission from Elsevier).

Kim et al. [61] studied and compared two systems to prepare cellulose solutions for electrospinning. They compared LiCl/DMAc and NMMO, and they also studied the influence of coagulation vs. air drying on the morphology of fibers. Several important conclusions were pointed out in this work. First, the most uniform fibers were obtained when a coagulation bath was used, wherein the removal of solvents from fibers is more efficient. The use of LiCl/DMAc required heating the collector to obtain dry fibers, while the nozzle and coagulation bath were at room temperature (RT) (Figure 4). Using the system NMMO/water, the solution of cellulose needed to be heated at 70–110 °C prior to spinning while the collector could be kept at RT, and the coagulation bath was cooled to 9–10 °C. The fibers obtained from both systems had diameters within the range 250–750 nm. Additionally, fibers obtained from the LiCl/DMAc system were mostly amorphous, while fibers from the NMMO/water system had a crystallinity between 40 and 60% depending on the electrospinning processing conditions [61].

Magalhaes et al. [62] used coaxial electrospinning to produce all cellulose nanocomposite comprised of cellulose nanofibers as the outer shell and CNCs (obtained by acidic hydrolysis) as the core material. A cellulose solution used for the shell of the fibers was prepared by dissolving cellulose in NMMO at 120 °C; later, the solution was diluted with DMSO. For the core of the complex fibers, cotton fibers were subjected to acid hydrolysis, followed by solvent exchange from water suspension to DMSO suspension of CNCs. As a collector, an aluminum screen was used, which was immersed in a coagulation bath consisting of cooled water (10 °C). The results showed that the CNCs were well dispersed in the cellulose matrix, producing fibrous films in which individual fibers of diameters in the range of 300–800 nm were produced.

Ohkawa et al. [38] reported the preparation of pure cellulose nanofibers via electrospinning at room temperature without the use of a coagulation bath and using dissolved cotton and wood pulp (average Mw 36,000–40,000) in trifluoroacetic acid (TFA). They used a variety of solution concentrations (2–5 wt%) and found that 2 wt% and 3 wt% were too low to produce nanofibers, while 4 wt%, 4.5 wt%, and 5 wt% concentrations resulted in nanofibers 70–130 nm in diameter. Additionally, acetylsalicylic acid or nicotine was added to a cellulose–TFA solution prior to spinning to produce drug-loaded nanofibers. The addition of these substances (drugs) influenced the morphology of spun fibers in terms that occasionally beaded structures were present on electrospun fibers, thus suggesting that acetylsalicylic acid causes cellulose macromolecules to aggregate, while in the case of nicotine-loaded fibers, this effect was less pronounced.

Li et al. [63] used modified electrospinning apparatus to produce nanofibers from high-molecular-weight cotton. Authors proposed that instead of heating the needle tip or collector to obtain high-quality cellulose nanofibers, the pathway taken by the solution between the tip of the needle and the collector should be heated with the help of an infrared lamp. The orientation of the nanofibers was also conditioned by the rotation speed of the collector, increasing the preferential orientation of the fibers by increasing the rotational speed to 1500 r/min. Cellulose solutions for electrospinning were prepared by dissolving cellulose in LiCl/DMAc, preceded by steps that improve dissolution, i.e., soaking and swelling cellulose in water, washing with methanol, and double solvent exchanges steps with DMAc. The concentration of cellulose in solution varied from 1 to 1.35 wt%, and the resulting electrospun nanofibers had a diameter between 150 and 300 nm.

Later, Li et al. [39] used the same device to produce cellulose nanofibers, from 1.15 wt% solution in LiCl/DMAc to subsequently functionalize them with cerium dioxide to impart UV protective properties. In fact, these authors reported that the functionalized material exhibited much higher UV absorbance than non-functionalized cellulose nanofibers.

In the work by Otsuka et al. [64], the authors used softwood and hardwood pulp with the addition of tunicate to prepare ultrafine nanofibers with diameters ranging from 40 nm to 96 nm. The mixture of solvents (trifluoroacetic acid and dichloroethane) was used with a concentration of pulp between 1.3 wt% and 1.9 wt%.

**Figure 5:**
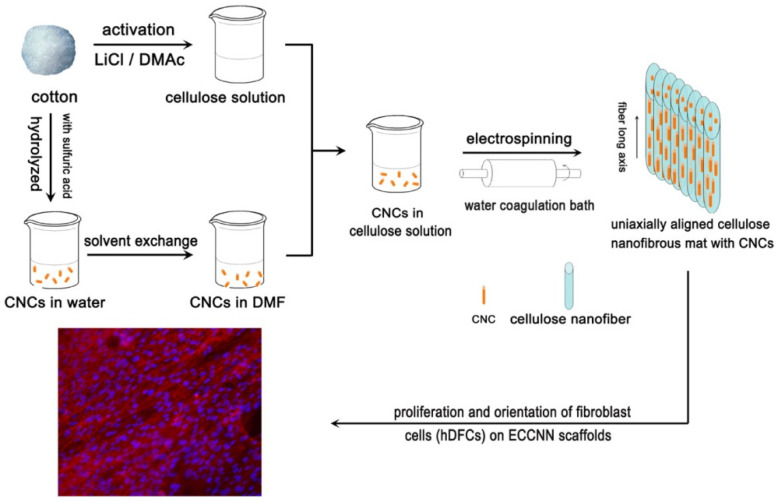
A scheme of the preparation of all-cellulose composite nanofibers from the same source using electrospinning and its potential use as scaffolds (reprinted with permission from He, X.; Xiao, Q.; Lu, C.; Wang, Y.; Zhang, X.; Zhao, J.; Zhang, W.; Zhang, X.; Deng, Y. Uniaxially aligned electrospun all-cellulose nanocomposite nanofibers reinforced with cellulose nanocrystals: Scaffold for tissue engineering. *Biomacromolecules* **2014**, *15*, 618–627. Copyright (2014), American Chemical Society).

He at al. [40] prepared an all-cellulose composite membrane from cotton cellulose (Figure 5). First, they used cotton fibers to prepare CNCs using the standard acid hydrolysis procedure. The CNCs were 150−250 nm in length and 15−25 nm in diameter and were suspended in DMF. After the dissolution of another portion of cotton cellulose in LiCl/DMAc, CNCs suspended in DMF was added to the cellulose solution.

The resulting electrospun nanofibers had an average diameter of 220 nm. TEM analysis indicated the alignment of CNCs along the long axis of the nanofibers. The introduction of CNCs in the cellulose nanofibrous membrane increased the tensile strength and Young’s modulus by more than 100%. The addition of CNCs also increased the thermal stability. The authors proposed the use of all-cellulose membranes as scaffolds and artificial blood vessels.

The use of volatile and sometimes toxic solvents remains a challenge for electrospinning of cellulose fibers. This should be addressed in the future due to a world orienting towards eco-friendly and green technologies. Recently, Hell et al. [65] proposed an eco-friendly procedure for the preparation of cellulose nanofibers. Since cellulose is not soluble in water, before electrospinning, cellulose is oxidized to dialdehyde cellulose and subjected to electrospinning in combination with polyvinyl alcohol using only water as a solvent. Dialdehyde cellulose alone cannot be spun, but in combination with PVA, nanofibers with diameters in a range of 200–500 nm were produced. This result allows the inference that the oxidation and functionalization of cellulose before electrospinning can be used to alter the solvent system used to make the cellulose nanofibers, thus making the process more eco-friendly.

### 3.2. Dry Spinning

**Figure 6:**
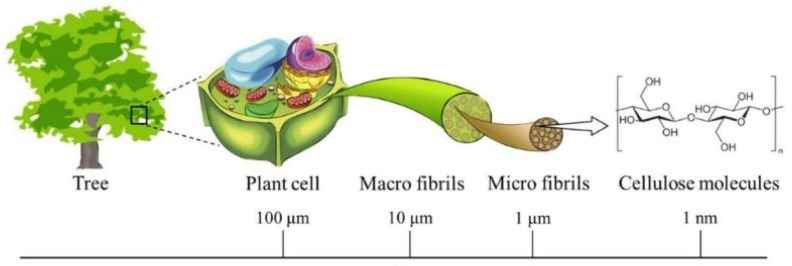
Cellulose contained in plants has a hierarchical structure from the meter to nanometer scale (reprinted with permission of authors from reference [67]).

Besides electrospinning, there are several other methods that have been extensively used to process cellulose nanofibers, namely dry spinning, wet spinning, and combined dry-jet wet spinning. These techniques have mostly been used to convert CNFs into filaments, as presented in Table 2. The isolation of CNFs and CNCs has been utilized considering the fact that natural cellulose fibers are comprised of micro fibrils [19,20,21,66] which can be isolated in the nanometer range (Figure 6) [67]. CNFs are usually isolated from various sources using either mechanical processing or physicochemical routes. CNFs are nanofibers with diameters of 5–100 nm and lengths ranging from 0.5 to several micrometers, containing high crystallinity [19]. CNCs (also called nanocrystalline cellulose or cellulose nanowhiskers) are most frequently obtained from cellulose using acidic hydrolysis, whereby amorphous parts of cellulose are destroyed, leaving highly crystalline (54–88%) whiskers of 5–50 nm in diameter and 100–500 nm in length. In order to produce usable membranes and filaments from CNFs, dry and wet spinning techniques to spin filaments have been proposed as successful routes for reassembling isolated CNFs into usable materials, as will be highlighted in this review.

Dry spinning can be used on isolated nanofibrils (CNFs) by inducing them to be oriented to finally generate strong filaments [41,42]. In the work [43], the authors first isolated CNFs from bleached banana rachis pulp, utilizing mechanical grinding. In the first step, 2 wt% suspension was converted to hydrogel, and different resultant concentrations (8%, 10% and 12%) of spinning dope were achieved by gel concentration using several centrifugation steps. Spinning dope of a desired concentration was fed to a capillary rheometer, and filaments were manually spun using different spinning rates and collected on a glass plate. The mechanical properties of the produced filaments showed an increasing value with increasing spinning rate and decreasing CNF concentration in a spinning solution. Moreover, the authors concluded that spun filaments have mechanical properties similar to the regenerated viscose filaments (11 GPa), pointing out that CNF filaments were spun from biomass waste using a simple route, thus having high environmental and economic potential.

Hooshmand et al. [44] later added hydroxyethyl cellulose (HEC) to assist the spinning of cellulose filaments using the same technology, utilizing a capillary rheometer. The HEC increased the wet strength of the dope, allowing stable spinning at low CNF concentrations. Moreover, the addition of HEC and cold drawing post-spinning improved the orientation of the CNFs in the filament, resulting in enhanced tensile strength and modulus by more than 70% as well as decreased hydrophilicity.

Shen et al. [45] used CNF suspension, prepared from bleached birch Kraft pulp, for the preparation of continuous CNF filament with an average diameter of 16 μm. The filaments were collected on a rotating low-friction surface (polyethylene-covered capstan), which, as the authors claimed, prevented shrinkage during drying of the as-spun filaments. Moreover, the mechanical properties and orientation of CNFs in the filament were controlled by controlling the spinning rate and rotational speed of the collector.

Ghasemi et al. [46] investigated the influence of the drying temperature on dry spinning of cellulose filaments from CNFs. The heating gun was placed above the collector to provide continuous drying of the produced filament. The temperatures for drying the spun filaments ranged from room temperature to 430 °C. The authors concluded that there was no statistically significant difference in mechanical properties between filaments that were dried at higher temperatures.

Ghasemi et al. [42] also studied the influence of different substrates for the collector on the morphology of spun cellulose nanofilaments. Teflon tape, Teflon film, and glass were used as the collector materials. Additionally, oil was added as a friction modifier in the form of a thin and thick layer on the collector. It was found that when a thick layer of oil was applied to the investigated substrates, spun filaments had the best cylindrical shape.

### 3.3. Solution Blow Spinning

Solution blow spinning is a relatively novel technique for the preparation of nanofibers, which was introduced in 2009 by Medeiros et al. [68] as an alternative to electrospinning. The main advantage of SBS over electrospinning, ES, is its higher production rate of nanofibers and films of similar properties to those generated by ES, whereby practically all types of solvents can be used [68]. In SBS, a syringe pump delivers a polymer solution to a system of concentric nozzles where the polymer solution enters through the inner nozzle while a constant, high-velocity gas flow is sustained through the outer nozzle to finally help the solution ejection at the nozzle exit and the evaporation of the solvent. During spinning, the solvent rapidly evaporates, forming fibers that, when they reach a solid substrate or the collector, may lead to a kind of web or mat depending on the conditions of the process. The collector is usually a rotating drum located at a previously set distance from the nozzle (working distance). SBS was successfully used to prepare various polymers in the form of mats or films: poly(methyl methacrylate) PMMA [69], poly(lactic acid) PLA [68], polystyrene PS [70], polyaniline [68] and composite PLA/CNCs [71], polyvinylidene fluoride PVDF [72,73], and poly(ethylene-co-vinyl acetate) EVA [74].

There are several reports on using SBS to produce cellulose fibers; however, the resulting fibers have diameters mostly above 1000 nm [47,48,49].

The solution blow spinning technique has been utilized in a production of cellulose nanofibers from wood pulp [47]. A 2 wt% solution of cellulose in 8% LiCl/DMAc was employed in an SBS device where fibers passing from the nozzle to the collector were heated along the path. However, the authors pointed out that so-called the spinning cabinet around the path from the nozzle to the collector was heated rather than using air at room temperature; therefore, a gradient of temperature was created, a direct consequence of which is that there is no way to control the temperature along the spinning line. In other words, the exact temperature during spinning remained uncertain. The authors offered an estimated spinning temperature based on the measured temperature near the collector, i.e., the end of the spin line [47]. The resulting nanofibers had an average diameter of 260–1900 nm.

**Figure 7:**
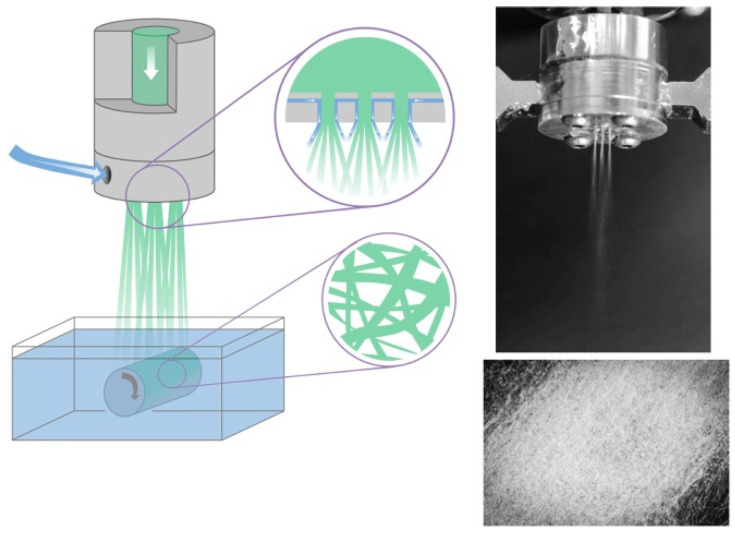
Dry-wet solution blow spinning of cellulose fibers from solution in ionic liquid (reprinted from reference Jedvert, K.; Idström, A.; Köhnke, T.; Alkhagen, M. Cellulosic nonwovens produced via efficient solution blowing technique. *J. Appl. Polym. Sci*. **2020**, *48339*, 1–9).

A few years later, Jedvert et al. [48] used dry-wet solution blow spinning (Figure 7).

Dissolved cellulose pulp in ionic liquid 1-ethyl-3-methylimidazolium acetate (EMIMAc) was spun into fibers from a wide range of concentrations while fibers were collected on a rotating cylinder submerged in water. Before SBS, the cellulose solution was heated to a certain temperature (65–95 °C). The resulting fibers were in the micrometric range (2–20 μm of diameter).

**Figure 8:**
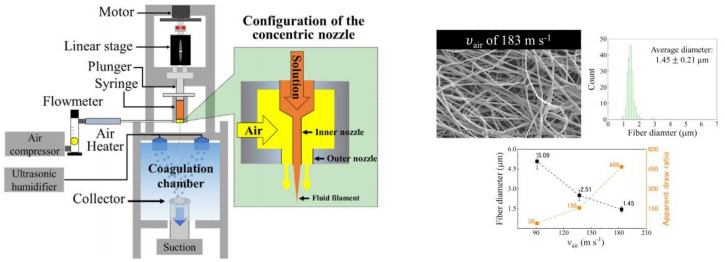
Schematic representation of wet solution blow spinning apparatus of cellulose fibers with mist coagulation chamber (left) and dependency of fiber diameter on air velocity during solution blow spinning (reprinted from the *European Polymer Journal*, **2020**, *125*, 109513, Zhang, J.; Kitayama, H.; Gotoh, Y. High strength ultrafine cellulose fibers generated by solution blow spinning, with permission from Elsevier).

Zhang et al. [49] in their work changed the solvent for cellulose pulp: instead of EMIMAc, they used 1-ethyl-3-methylimidazolium diethyl phosphate (EMIMDEP) and successfully spun cellulose using the dry-wet solution blow spinning technique from 5 wt% solution. Both spinning dope and air were heated, while the collector was placed in a water-mist-filled chamber (Figure 8). The resulting fibers had smaller diameters compared to the ones obtained by Jedvert et al. [48], ranging from approximately 1.4 to 5 μm; however, they were still not in the nanometric range. Another interesting observation made by Zhang is that the diameter of fibers was dependent on the air velocity during spinning, as seen in Figure 8 The air flux at the exit of the nozzle induces a certain deformation of polymer fluid and provides a draw ratio of the fiber jet during exit. With low air velocities (46 m s^−1^), the authors found that the diameter of fluid at the exit of the nozzle was higher than diameter of nozzle itself, implying that the drag force of air was too low to overcome elastic recovery of the extruded fluid, which ultimately led to nanofibers with higher diameters. By increasing the air velocity, the fluid becomes more stretched, resulting in thinner fibers; however, when the air velocity reaches 183 m s^−1^, a pullout effect occurs, resulting from solution detaching from the spinneret, which breaks the polymer jet and makes processing unstable. All of these results and observations indicate that solution blow spinning, as a technique, still remains a challenge for the production of cellulose-based nanofibers.

### 3.4. Wet Spinning

Wet spinning, the opposite of dry spinning, uses a bath where spun filaments coagulate, forming fiber before drying. This is one of the most utilized procedures for the preparation of cellulose fibers. The spinneret is usually immersed in the coagulation bath, and fibers are formed immediately upon exiting the spinneret.

Zhu et al. [50] produced wet-spun nanofibers with an average diameter of 25 nm from cotton pulp. The cotton pulp was dissolved in the system NaOH/LiOH/Urea/H_2_O resulting in spinning dope of 5.8 wt% concentration. The coagulation bath contained 15 wt% phytic acid and 5 wt% sodium sulfate aqueous solution at a temperature of 5 °C. The resulting multifilament material showed excellent mechanical properties with fiber strengths of 3.5 cN/dtex and 2.5 cN/dtex in dry and wet state, respectively.

**Figure 9:**
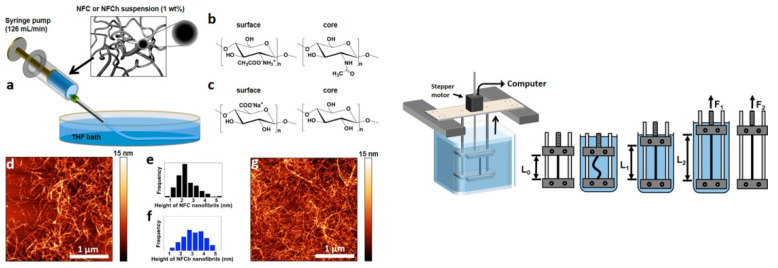
(**left**) Wet spinning of CNFs. (**a**) Wet-spinning setup using controlled extrusion with a syringe pump into a THF coagulation bath using NFC and NFCh dispersions, containing surface modifications as depicted in (**b**) and (**c**). (**d**–**g**) AFM height images for NFC (**d**) and NFCh (**g**), as well as their corresponding image analysis concerning the average height of 200 nanofibrils for each class of nanoparticle ((**e**) = NFC; (**f**) = NFCh); (**right**) schematic drawing for the computer-controlled wet-stretching device into which a fiber can be clamped and immersed into a liquid and stretched using controlled strain rates (reprinted with permission from Torres-Rendon, J.G.; Schacher, F.H.; Ifuku, S.; Walther, A. Mechanical performance of macrofibers of cellulose and chitin nanofibrils aligned by wet-stretching: A critical comparison. *Biomacromolecules* **2014**, *15*, 2709–2717. Copyright 2014, American Chemical Society).

Recently, authors [51] have been using wet spinning for the production of filaments from CNFs. The same technique was used for nanofibrilated chitin, another polysaccharide that is very abundant in nature and has high application potential. In addition to wet spinning, authors have applied wet stretching in order to reinforce the produced filament (Figure 9).

The resultant filaments are prepared from a suspension of 1 wt% CNFs (isolated by TEMPO-oxidation of softwood pulp) in distilled and deionized water. After spinning and wet stretching, the mechanical stiffness increased by fourfold due to better orientation and alignment of the CNFs in the spun filament.

Geng et al. [52] used wet spinning for a production of filaments from a 1 wt% suspension of CNFs into acetone in a coagulation bath. They studied the addition of polyamide-epichlorohydrin (PAE), which behaves as a wet strengthening agent in the paper industry. Interestingly, in order to prepare a mixture of CNFs and PAE, a 1 wt% suspension was diluted to 0.3 wt% with deionized water. They used a CNF-to-PAE ratio of 10:0.5 and 10:1. After mixing, by heating the suspension to 40 °C, they restored the original intended concentration of suspension 1 wt%. CNFs were originally prepared by TEMPO-oxidation and mechanical disintegration of raw jute fibers, wherein nanofibers with an average width of 5.4 nm and length of 400 nm were isolated.

### 3.5. Hybrid Dry-Jet Wet Spinning

**Figure 10:**
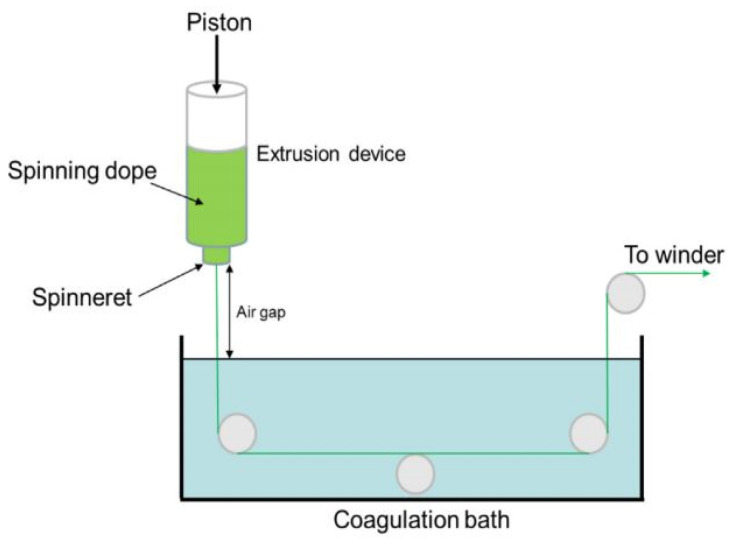
General scheme of dry-jet wet spinning using air gap between spinneret and coagulation bath (reprinted from *International Journal of Biological Micromolecules*, **2016**, *92*, 1197–1204, Boy, R.; Narayanan, G.; Chung, C.C.; Kotek, R. Novel cellulose-collagen blend biofibers prepared from an amine/salt solvent system, with permission from Elsevier).

Dry-jet wet spinning is a technique that is essentially similar to wet spinning, but an air gap is added between the spinneret and the coagulation bath (Figure 10) [53].

**Figure 11:**
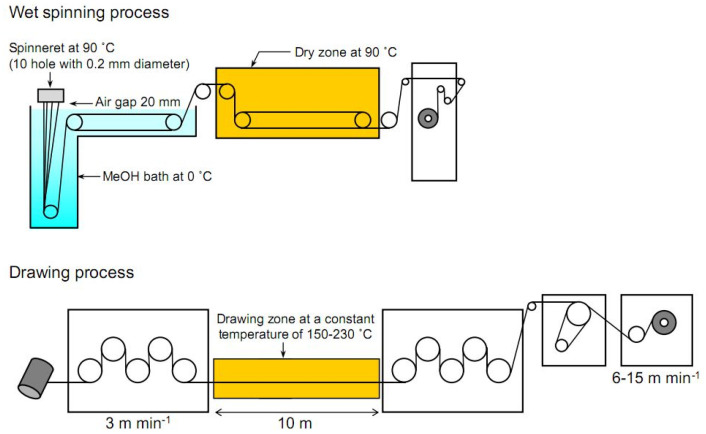
Scheme showing all stages during dry-wet spinning process (reprinted from *Polymer*, **2013**, *54*, 935–914, Endo, R.; Saito, T.; Isogai, A. TEMPO-oxidized cellulose nanofibril/poly(vinyl alcohol) composite drawn fibers, with permission from Elsevier).

Endo et al. [54] used wet spinning with a small air gap between spinneret and coagulation bath for a production of hybrid filament composed of PVA filled with TEMPO-oxidized nanofibrils (TOCN). The prepared hybrid filament exhibits a high tensile modulus, reaching a value of 57 GPa. The proposed wet spinning and drying scheme is given in Figure 11.

Dry-jet wet spinning was used by Song et al. [55] to produce bionanocomponent fiber from microcrystalline cellulose (MCC) with addition of nano SiO_2_. The solvent for microcrystalline cellulose was 1-allyl-3-methylimidazolium chloride (AMIMCl) ionic liquid. Silica particles were of 80 nm diameter. For spinning, 5 wt% MCC solutions were used with different ratio of nano SiO_2_ (0.2–0.6%). The air gap was 3 cm and the coagulation bath contained deionized water. After, the spinning fibers were vacuum dried for 48 h. Authors reported good dispersion of SiO_2_ in cellulose fibers and an increase in thermal stability for the composite fibers.

Kafy et al. [56] used the so-called ion-mediated wet spinning (IMWS) of CNFs into long filaments where 5 wt% CaCl_2_ solution was used in the coagulation bath. Spinning dope consisted of 2 wt% suspension of CNFs prepared from TEMPO oxidized hardwood bleach pulp. The eventual cellulose crosslinking with the Ca^2+^ ions of the coagulation bath enabled the production of very-high-strength cellulose fibers.

### 3.6. Interfacial Polyelectrolyte Complex Spinning

**Figure 12:**
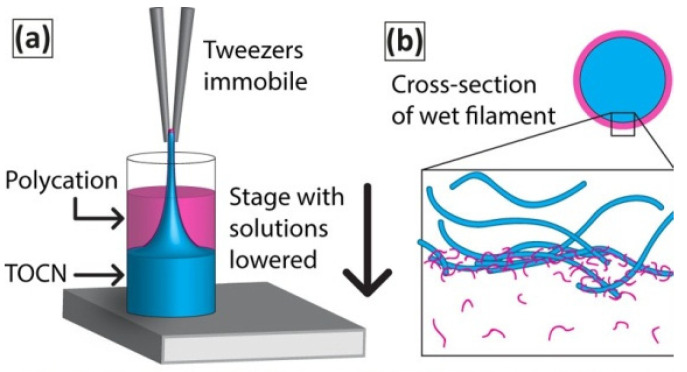
Schematic representation of (**a**) process of IPC of TOCN/polycation fibers, and (**b**) suggested cross-section of formed wet filament with polycation containing solution on the periphery and the formed polyelectrolyte complex membrane at the interface of the two fluids (Reprinted from reference Toivonen, M.S.; Kurki-Suonio, S.; Wagermaier, W.; Hynninen, V.; Hietala, S.; Ikkala, O. Interfacial Polyelectrolyte Complex Spinning of Cellulose Nanofibrils for Advanced Bicomponent Fibers. *Biomacromolecules* **2017**, *18*, 1293–1301, Copyright 2017, American Chemical Society).

Interfacial polyelectrolyte complex (IPC) spinning of cellulose nanofibers [57] is based on a spontaneous formation of continuous filament upon mechanical drawing of the interface formed by two solutions of oppositely charged polyelectrolytes. The advantage of IPC as the medium used for the process is that it is aqueous and eco-friendly, in addition to the ability of spinning multicomponent filaments. Toivonen et al. [57] prepared TEMPO-oxidized cellulose nanofibrils, TOCN, by spinning anionic TOCN with the polycation poly(diallyldimethylammoniumchloride) (PDADMAC) and chitosan (Figure 12).

The successful preparation of multicomponent fibers of circular shape was reported. The importance of IPC spinning, as the authors stated, is the fact that, in IPC, there is no shear stress induced upon the CNF gel, contrary to what occurs when gel flows through a needle or other type of capillary. In this way, fibrils are oriented by lateral contraction during drying and by self-assembly at the interface. However, the authors proposed that the combination of IPC with shear-induced orientation prior to stretching might lead to further anisotropy of mechanical properties.

For biological application and mimicking muscle filaments, IPC proved to be very effective, especially for spinning CNFs with antimicrobial chitosan into ordered structures that might be used as biomaterials [58]. As another completely different potential application, the combination of CNFs and graphene oxide with chitosan was used to produce graphene fiber-shaped electrodes for supercapacitors [59].

## 4. Spinning of Cellulose Derivatives

### 4.1. Cellulose Esters

Cellulose acetate is the most important ester of cellulose. Commercial cellulose acetate has substituted OH groups in cellulose by acetyl groups, and its degree of substitution is approximately 2.5. The derivative for which all cellulose hydroxyl groups are substituted by acetyl groups is called triacetate (CTA). Partial hydrolysis up to DS 2.4–2.5 after acetylation yields to commercial cellulose diacetate, also simply referred to as acetate [17,75]. Commercial cellulose acetate has found its main applications in the production of textile fibers, highly transparent films, cigarette filters, eyewear, etc. Cellulose acetate is easily soluble in many organic solvents such as acetone, DMF, DMAc, chloroform, etc. Therefore, its use for spinning fibers from solutions is highly productive [17]. CTA and CA are both high-melting, high-strength hydrophobic polymers, with high UV stability, low inflammability and film transparency [75]. Even though acetates are melting derivatives of cellulose, required temperatures for their melting leads to partial degradation; therefore, melt spinning is not used for the production of CTA and CA fibers.

Commercial CA fibers are spun using conventional dry-spinning technology where a spinning dope made of 25% CA solution in acetone is used and the filament from spinneret is passed through a 4–6 m long heating column. On the other hand, CTA is usually spun from the solvent system methylene chloride/methanol, even though CTA is more often used to make films than fibers [75] since it allows obtaining highly dimensionally stable films with low inflammability. Due to its exceptional properties, it is not surprising that cellulose acetate is the most used precursor for obtaining cellulose-based nanofibers.

Cellulose acetate nanofibers are usually prepared using electrospinning, rotary jet spinning, wet spinning, and solution blow spinning, as outlined in Table 3.

For some techniques such as solution blow spinning, pure CA nanofibers preparation is still a challenge and, up until now, CA nanofibers were successfully spun only alongside other polymers such is PAN [78]. On the other hand, wet spinning of CA can be used in coaxial configuration, alongside CNFs, to produce mechanically strong and highly water-absorptive filaments [41,81,82,83]. Currently, electrospinning is by far the superior technique for the production of cellulose acetate nanofibers, even though it has its own disadvantages, for example, long production times and the use of high-voltage electrical fields, which could be addressed in the future by exploring other techniques for CA nanofiber preparation, as outlined in Table 3.

Electrospinning was utilized in cellulose acetate for the first time in 2002 [22]. Two decades later, an impressive amount of work has been published using cellulose acetate in various solvents (Table 4) and considering several concentrations of spinning dope to obtain nanofibers of various shapes and sizes, with diameters ranging from 60 to 5000 nm. Usually, a very high concentration of spinning solution is used, preferentially above 15 wt%, in order to obtain beadless smooth nanofibers, while other electrospinning conditions and solvents used mainly influence the resulting diameter of nanofibers. A threshold concentration of 15 wt% has been reported as being appropriate for obtaining smooth cellulose acetate fibers; however, it is important to highlight here that most investigations rely on using acetate of Mn 30,000. In fact, the molecular weight of the polymer can be an important factor of the final morphology of the electrospun acetate. As can be seen in Table 1, Santos-Sauceda et al. [84] obtained smooth fibers with an average diameter of approximately 1000 nm from 8 wt% solution of CA in a mixture of acetone and water (80:20), but they were using cellulose acetate of higher molecular weight Mn 50,000. However, when they used 4 wt% and 6 wt% solutions, fibers with beads and ribbons were produced. These results suggest that the molecular weight of cellulose acetate must condition the threshold concentration for obtaining smooth fibers during electrospinning.

In addition to the polymer molecular weight, the solvent system highly influences the production of cellulose acetate nanofiber. It has been shown that the solvent system can be crucial for the spinnability of the polymer solution [77,110], not just in terms of the solvent combination (nature of solvents) but also the composition of the solvent mixture as well. For example, Liu and Tang [96] showed that smooth cellulose acetate fibers can be obtained by using acetone/DMAc in a ratio of 2:1, but not vice versa. When using acetone/DMAc 1:2, fibers with beads were obtained. Generally, the use of only acetone is not recommended due to the high evaporation rate of acetone at room temperature, which can induce nozzle clogging during spinning.

Lee et al. [77] studied the effect of different ratios of DMF and acetone (6:4, 4:6, and 2:8) and concluded that higher proportion of DMF requires a higher polymer concentration in order to produce smooth fibers. It is also interesting to point out that the same cellulose acetate (Mn 30,000) was used in both DMF/acetone mixture and dichloromethane/acetone mixture with a ratio of solvents of 3:1. As reported, to obtain smooth fibers from solution in DMF/acetone, the authors needed to use at least 17 wt% of polymer, while for solutions in DCM/acetone, a lower concentration was required (7.5 wt% and 10 wt%). Interestingly, the authors also pointed out that those solutions in the DCM/acetone system were harder to spin, obtaining thicker but highly porous fibers.

There are several reports of using system comprising of three components of solvent [76,114,119,122]. An especially interesting approach was provided by Hardick et al. [76], studying electrospining of cellulose acetate nanofibers from 20% solution in a mixture of three solvents, acetone/DMF/ethanol (2:2:1), under different spinning temperatures (17.5–32.5 °C) and humidities (20–70%). They concluded that increasing the air temperature during spinning reduces the average diameters of nanofibers while increase the humidity increases the fiber diameters. The authors claim that increased humidity also leads to better-quality beadless fibers, which indicates that both the temperature and humidity may influence the resulting fiber morphology. In addition, melt enthalpy was also affected by processing conditions, and it was reduced with increase of spinning temperature and increased by increasing the humidity during electrospinning.

**Figure 13:**
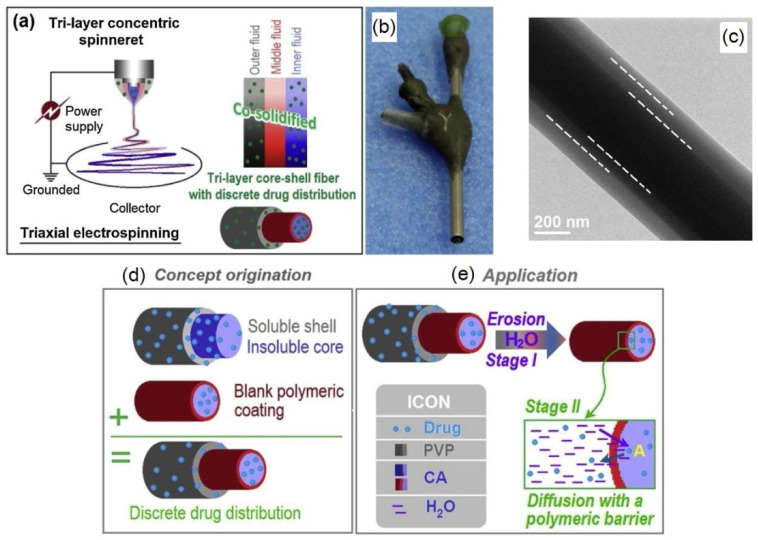
A scheme of triaxial electrospinning (**a**); triaxial spinneret (**b**); tricomponent fiber (**c**); scheme of the concept for triaxial spinning (**d**); application of tricomponent fiber for sustainable drug release (**e**) (reprinted and adapted from *Carbohydrate Polymers*, **2020**, *243*, 116477, Yang, Y.; Chang, S.; Bai, Y.; Du, Y.; Yu, D.G. Electrospun triaxial nanofibers with middle blank cellulose acetate layers for accurate dual-stage drug release, with permission from Elsevier).

Recently, Yang et al. [122] reported a novel approach to prepare coaxial tricomponent nanofibers by electrospinning for controlled drug release. Water-soluble PVP with ketoprofen was used as an outer layer while CA/KET was used as the fiber core and blank CA was used in a middle, which provided better drug release profiles with a longer time period of sustained release in the second stage compared with the two-component coaxial spun fibers (Figure 13). The reported time period for releasing 90% of the drug in the second stage for tricomponent coaxial fibers is 60.7 h, compared with 35.8 h from the simple core-shell fiber.

Application of electrospun cellulose acetate is very diverse, ranging from the already-mentioned drug delivery to many more, as outlined in Table 5. Besides its biomedical application, which implies the use of drugs and other antibacterial agents, electrospun cellulose acetate, with the addition of other substances, can be used in sensors, filtering membranes, high absorption materials, or separatory membranes (e.g., to separate oil and water, as proposed in [124]). As indicated in Table 5, there is a possibility to include functional agents prior or post-spinning. Sometimes, a combined approach, using both methods, is followed to obtain highly functional material, where one substance is added prior to spinning, and then additional functionalization is performed after spinning. An especially interesting approach regarding post-spinning treatments is deacetylation of the produced cellulose acetate membrane to obtain pure cellulose nanofibrous membranes. Its spinnability, variety of available solvents, and established procedures make cellulose acetate the cellulose derivative of choice to be used and employed in nanofiber production. However, often for some applications, there is a demand for pure cellulose nanofibrous membrane. Therefore, many authors have decided to produce membranes from cellulose acetate, using already-familiar and established protocols, and subsequently regenerate pure cellulose using simple hydrolysis of acetate to OH groups.

**Figure 14:**
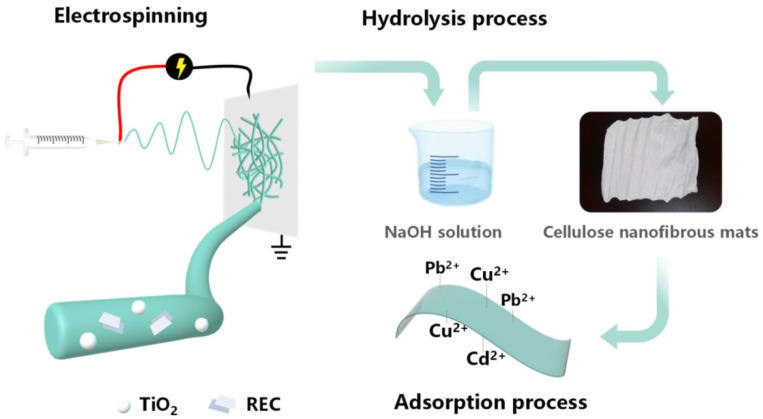
Schematic diagram of preparation of cellulose membrane via electrospinning of cellulose acetate with TiO_2_ nanoparticles and rectorite, followed by deacetylation to pure cellulose with increased absorption properties towards heavy metal ions Pb^2+^, Cu^2+^ and Cd^2+^ (reprinted from *International Journal of Biological Macromolecules* **2021**, *183*, 245–253, Wang, C.; Zhan, Y.; Wu, Y.; Shi, X.; Du, Y.; Luo, Y.; Deng, H. TiO_2_/rectorite-trapped cellulose composite nanofibrous mats for multiple heavy metal adsorption, with permission from Elsevier).

Hydrolysis can be performed using aqueous NaOH or NaOH/ethanol solutions [22,86,99,102,109,141]. The scheme of hydrolysis of CA to obtain pure cellulose nanofibrous membranes for its potential use as a material for heavy metal removal is given in Figure 14 [126].

Additionally, after hydrolysis, the nonwoven membrane can be further treated with acetone [142] in order to ensure complete removal of non-hydrolyzed parts.

A significant decrease in deacetylation times can be achieved by using ultrasonication during hydrolysis in aqueous NaOH and NaOH/ethanol solutions, reducing the times of the deacetylation process from 30 h to just 1 h [91]. Regenerated membrane is usually more hydrophilic with a higher water absorption capacity [22], has a more porous structure, which is desirable for molecular interaction [109], or can serve as a material for further functionalization [107,143]. As mentioned before, OH groups of cellulose are susceptible to transformation and functionalization. The regenerated cellulose membrane can be used as a catalytic membrane [104], sensor for environmental contaminants [143], and immobilization surface for enzymes [88,107,144], as well as for the removal of heavy metal ions [126].

Needleless electrospinning has becoming more popular in recent years due to the fact that the production rate with needleless ES is much higher compared to classic needle electrospinning [145]. During needleless electrospinning, the polymer solution is pumped through a special type of spinneret whereby multiple jets can be formed during electrospinning, thus increasing the production and providing the possibility to obtain a very large nanofibrous membrane (e.g., 1.1 m × 1.7 m) [145].

Wu and coauthors [121] proposed the use of a metal-plate needleless spinneret to produce cellulose acetate nanofibers. In that work, using various solvent systems (acetone/DMAc, acetone/DMSO and acetic acid/water), the authors compared the needleless cellulose acetate output with that obtained with classical needle ES and concluded that the most promising solvent system was acetone/DMSO (2:1 *v*/*v*) with a processing rate of 10–20 mL/h. The concentration of CA in solution varied from 14 to 18 wt%, obtaining the best-quality smooth nanofibers when using 16 wt%. For comparison, a common solution processing rate in classical electrospinning with a single needle for some polymers was used (0.05 mL/h). In the work by Wu et al. [121], the production output of CA membrane with needle ES for 1 h was approximately 0.02 g of material, while with needleless ES apparatus it was 0.85 g/h. The diameter of the produced nanofibers was in the range 273–760 nm, depending on the polymer concentration and solvent system. However, comparing the outputs obtained with the same spinning solution on different setups, the average diameter of CA nanofibers can vary from 414 nm to 331 nm, depending on whether needle or needleless electrospinning is used, respectively [121].

Apart from the number of advantages of producing cellulose acetate nanofibers using electrospinning, it should also be highlighted that it can be used for recycling, which is important from the point of view of environmental challenges and reducing waste. For instance, Huang et al. [146] used cigarette filters as a starting recyclable material to produce a potent separator for Li-ion batteries using electrospinning of cellulose acetate. In this research cellulose acetate was extracted from waste cigarette filters and used in coaxial electrospinning with PVDF-HFP to obtain fibers with a core constituted by the CA. After electrospinning, the membrane was hydrolyzed, and CA was converted to pure cellulose, which has a higher thermal stability and hydrophilicity that, together with excellent electrochemical properties, made this CA-based membrane a very good candidate as a separator for Li-ion batteries.

With the same idea of recycling, Liu et al. [147] used old blue jeans as a cotton source to regenerate pure cellulose and prepare nanofibrous membrane using dry-wet electrospinning. With these examples, the potential of using both cellulose and cellulose derivatives in recycling and to add novel value to old products is clear.

**Figure 15:**
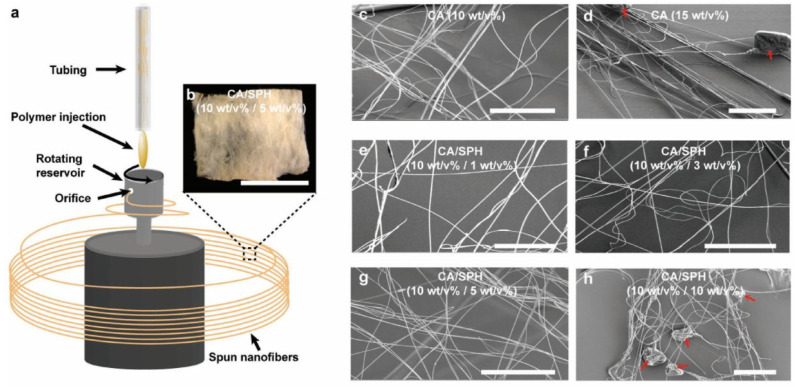
Scheme or rotary jet spinning (**a**); resulting cellulose acetate spun membrane (**b**); different morphology observed by SEM of fibers with various concentrations of CA and soy protein SPH (**c**–**h**) (reprinted from *Advanced Healthcare Materials*, **2018**, *7*, 1–13, Ahn, S.; Chantre, C.O.; Gannon, A.R.; Lind, J.U.; Campbell, P.H.; Grevesse, T.; O’Connor, B.B.; Parker, K.K. Soy Protein/Cellulose Nanofiber Scaffolds Mimicking Skin Extracellular Matrix for Enhanced Wound Healing, with permission from John Wiley & Sons, Inc.).

Besides widely used electrospinning, there are few other techniques for acetate nanofiber production, as indicated in Table 3. Ahn et al. [79] reported the preparation of nanofibers from a mixture of cellulose acetate and soy protein, SPH, using rotary jet spinning to produce scaffold with bioactive properties (Figure 15). The authors showed an increased water retention capacity of produced membrane with the ability to enhance proliferation of fibroblast, which can accelerate re-epithelialization and reduce scar formation and collagen anisotropy. Nanofibers were spun from a solution of CA and soy protein in hexafluoroisopropanol. The resulting nanofibers of pure CA were, on average, 300 nm in diameter, while CA/SPH hybrid nanofibers were nearly 400 nm in diameter.

For the preparation of cellulose acetate nanofibers, a hybrid technique was also used: electrostatic induction-assisted solution blow spinning [80]. A 17 wt% solution of commercial CA dissolved in acetone/DMAc was spun into nanofibers. The drawing force on the polymer jet at the nozzle exit arose from two contributions: high-velocity air flow surrounding the nozzle and applied voltage. Then, the resulting fibers were hydrolyzed with NaOH/EtOH to produce pure cellulose membrane. The diameter of CA nanofibers ranged from 150 to 1000 nm, while after deacetylation, the diameters of the cellulose fibers ranged from 200 to 800 nm.

The production of cellulose acetate nanofibers using only SBS without the assistance of electrostatic force remains a challenge which should be addressed in the future to simplify the process as much as possible. The first step toward processing cellulose acetate using SBS was taken by Dadol et al. [78], although they did not produce pure cellulose acetate since assisted spinning with polyacrilonitrile (PAN) was used, thus producing PAN/CA composite. Both CA and PAN were dissolved in the same solvent (DMF) using the same concentration (9 wt%), being able to produce nanofibers only when the proportion of CA in PAN was in the range of 50–65%; therefore, pure CA nanofibers could not be spun.

Conventional wet spinning of cellulose usually results in thick fibers within the micrometer range (>20 μm) [81,141], but it can be used for obtaining nanofibers of cellulose acetate with cellulose [82,148]. Cellulose acetate can be used as a carrier polymer in coaxial spinning to prevent filament breakup during wet spinning of CNFs [148]. For spinning pure CNF, acetone in a coagulation bath was used, while for CA, water was used for coagulation. When spinning CNFs/CA as the core shell, water was used as a coagulation fluid, whereby the CNFs in the core solution were 1 wt%.

### 4.2. Cellulose Ethers

Cellulose ethers are another class of cellulose derivatives which have found its use in the preparation of nanofibers for different purposes. The etherification of cellulose is an important derivatization route which yields a very broad class of derivatives with many potential applications [75]. Among many, the most frequently used ethers for the production of nanofibers are ethylcellulose [149,150,151,152,153,154], hydroxypropyl cellulose [155,156,157,158], hydroxyethyl cellulose [159,160,161,162,163], methyl cellulose [164], and carboxymethyl cellulose (CMC) [165]. The solubility of cellulose ethers is highly dependable on the degree of substitution. Alkylcelluloses in general have one or more hydroxyl groups substituted by alkyl groups, mainly methyl and ethyl. The hydrophobicity of alkyl cellulose increases with the length of the alkyl chain and degree of substitution (DS). It has been shown that, for example, nanofibrous membrane, composed of ethylcellulose and gelatin, exhibits hydrophobic behavior, with increased content of ethylcellulose in a composite [151]. Commercial ethyl cellulose with a DS above 2 is hydrophobic and thermoplastic, being able to be extruded into films. Both ethyl and methyl celluloses, with DS above 2, are soluble in organic compounds, such as ethanol, acetone, toluene, while with DS below 2, these cellulose ethers are soluble in water [75].

Carboxymethyl cellulose (CMC) is commercially the most important cellulose ether. Commercial CMC, with DS of less than 1 (from 0.3–0.9), is biodegradable, non-toxic, and usable as a food additive and in pharmaceuticals. In fact, the use of CMC in the particular form of nanofibers, especially for food packaging or medical application, is very common [166].

**Figure 16:**
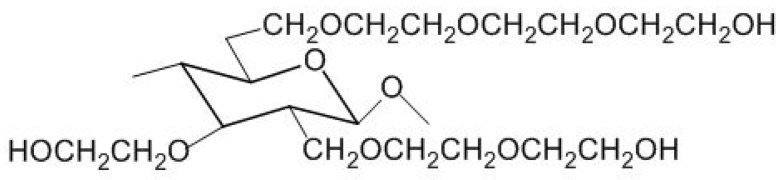
Example of the degree of substitution (DS) (e.g., 3) and molecular substitution (MS) (e.g., 6) on one anhydroglucose unit (AGU) of hydroxyethylcellulose.

For hydroxyalkyl ethers, such as hydroxyethyl cellulose (HEC) and hydroxypropyl cellulose (HPC), it is necessary to consider the so-called molecular substitution, MS (average number of alkylene oxide molecules per anhydroglucose unit, AGU as presented in Figure 16) in addition to the degree of substitution of hydroxyl groups on AGU, DS [75]. The usual ratio between these parameters is MS/DS = 1.5–2.5.

Most commercial HPC have an MS between 1.5 and 3, corresponding to values of DS between 0.8 and 1.2. An MS of 1 usually denotes water-soluble HPC. On the other hand, HPC is more hydrophobic than HEC. An HEC that is higher than MS is usually soluble in a mixture of water and some polar organic solvents, such as methanol. Moreover, it has been demonstrated that HPC has to have an MS of 4 to be soluble in cold water.

For cellulose ethers, the commonly used technique is electrospinning (Table 6). Moreover, cellulose ethers are frequently used in coaxial electrospinning, usually as a core material, to produce drug delivery nanofibrous membranes, whereby the slow, controlled release of drugs is possible thanks to the cellulose-based component [167,168]. Various coaxial configurations will be presented in detail in the next subsection.

Smooth electrospun fibers of HEC or HPC are difficult to obtain; therefore, HEC is mixed with polyvinyl alcohol [159,160,161] to produce fibers of submicronic size. Additionally, due to the water solubility of the produced material, crosslinking is usually performed with glutaraldehyde post-spinning [159,161] or photochemically during electrospinning [159]. The HEC nanofibrous membranes can have a potential application in tissue engineering since they are biocompatible and non-toxic [160]. Besides PVA, polyacrylonitrile (PAN) alongside HEC can be used [163] to produce nanofibers of 100–300 nm in diameter. As in the case of PVA/HEC, the PAN/HEC electrospun membranes must be crosslinked with glutaraldehyde to avoid water solubility for biomedical applications.

HPC is also electrospun usually alongside PVA [156,157,158] or PVP [157] to be used as a drug delivery system, as reported for diclofenac sodium [157] and papaverine hydrochloride [156].

#### Coaxial Electrospinning of Cellulose Ethers

**Figure 17:**
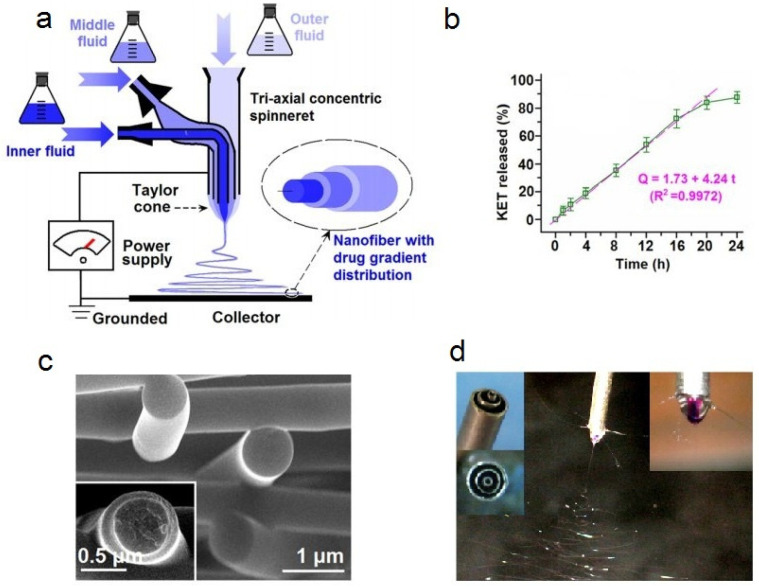
(**a**) Scheme of triaxial electrospinning; (**b**) kinetic profile of ketoprofen release over time from tiraxialy electrospun fibers; (**c**) cross sectional morphology of triaxial fibers and (**d**) digital photograph of triaxial electrospinning process (reprinted and adapted from *Applied Materials and Interfaces*, **2015**, *7*, 33, 18891–18897, Yu, D.G.; Li, X.Y.; Wang, X.; Yang, J.H.; Bligh, S.W.A.; Williams, G.R. Nanofibers Fabricated Using Triaxial Electrospinning as Zero Order Drug Delivery Systems, Copyright 2015, American Chemical Society).

Coaxial electrospinning is a one-step technique which enables the formation of multilayered fibers, a sheath-core, or even hollow fibers. This technique is especially convenient when the resulting fibers may have applications as antimicrobial drug delivery systems [170]. It is frequently used for electrospinning cellulose ethers. Triaxial electrospinning can be used to process ethyl cellulose loaded with different concentrations of ketoprofen for controlled drug release (Figure 17) [152].

The produced nanofibers had an average diameter of 750 nm; they were comprised of three layers of different concentration of ketoprofen following a particular trend in which there is a concentration increase from the outer to inner layer. Moreover, same authors showed the ability of the produced nanofibers to release ketoprofen over 20 h.

**Figure 18:**
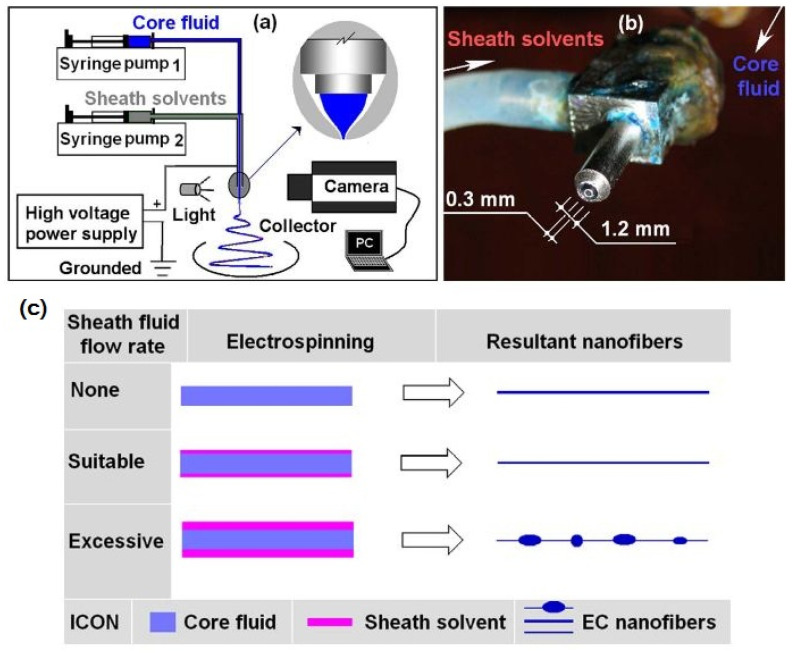
(**a**) Schematic diagram of modified coaxial electrospinning; (**b**) photograph of homemade concentric spinneret and (**c**) the proposed mechanism of nanofibers formation using sheath solvent in coaxial electrospinning (reprinted from reference [171]).

The work by Yu et al. [171] presented an innovative concept of coaxial electrospinning by using only solvent as sheath fluid (Figure 18). In this research, the sheath solvent flow rate influenced the morphology of electrospun ethylcellulose nanofibers. The core consisted of ethylcellulose dissolved in ethanol while pure ethanol was used as sheath fluid. The authors concluded that the proper selection of sheath flow, which allows matching the drawing process of the core fluid containing the polymer, could prevent blocking of the nozzle and controllable spinning of defined nanofibers.

Another approach used for processing ethyl cellulose is its spinning with polyvinylpyrrolidone (PVP) [172,173]. In [172], coaxial electrospinning was used utilizing PVP as the sheath polymer and EC as the core polymer. Ketoprofen was included in both polymer solutions, constituting a biphasic drug release system. For ethyl cellulose, pure ethanol was used as solvent with the addition of 3 wt% of ketoprofen, while for PVP, a mixture of DMAc and ethanol (1:9) with the addition of 1 wt% KET was used. The pure EC nanofibers spun from a 24 wt% solution had an average diameter of 710 nm, pure PVP spun from a 9 wt% solution resulted in nanofibers of 910 nm, and core-sheath fibers had diameters ranging from 780 to 940 nm depending on the flow rate of the sheath fluid. On the other hand, PVP provided faster release of drug while EC provided slower diffusion of drug from its structure. In another work carried out by Qian et al. [167], the same system of polymers was used, to which a different drug was added: acetaminophen.

**Figure 19:**
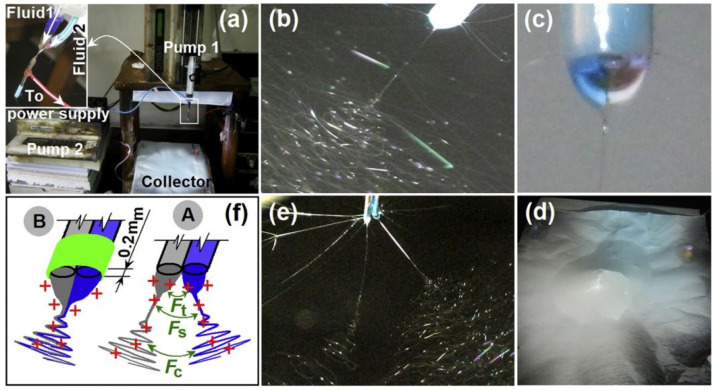
(**a**) Experimental apparatus of side-by-side electrospinning process; (**b**) photograph of typical side-by-side electrospinning process; (**c**) Janus Taylor cone formed with Teflon-coated spinneret; (**d**) mat of fibers produced using non-coated side-by-side spinneret; (**e**) separation of fluids when using non-coated side-by-side spinneret and (**f**) illustration of the influence of Teflon-coated spinneret: A separation of fluids arising from repulsive forces and B formation of the integrated Janus Taylor cone with the Teflon coating (reprinted from reference [174] with permission from Elsevier).

**Figure 20:**
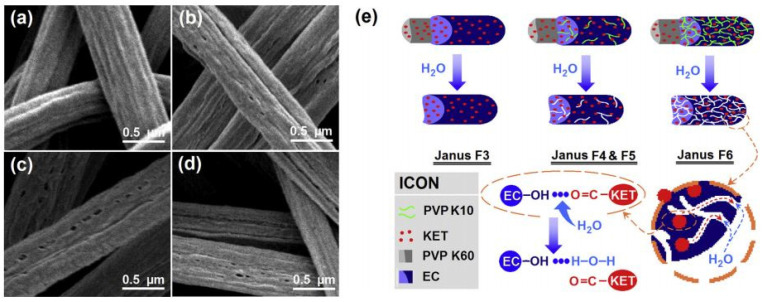
FESEM images of the fibers remaining after 24 h of dissolution (**a**–**d**) and proposed mechanism of drug release from Janus fibers (**e**) (reprinted from reference [174] with permission from Elsevier).

Yu et al. [174] used a side-by-side Teflon-coated spinneret for electrospinning PVP and EC loaded with ketoprofen, producing Janus fibers. The PVP was dissolved rapidly, delivering a loading dose of ketoprofen, while EC had a sustained release of drug (Figure 19 and Figure 20). In addition, these authors also included low-molecular-weight PVP in EC to better control and speed up the release of drug from the EC rich part of fiber. Single fibers of PVP or EC had average fiber diameters of 580 and 650 nm, respectively, while side-by-side fibers had almost double the diameter, showing a mean value of 1000 nm.

**Figure 21:**
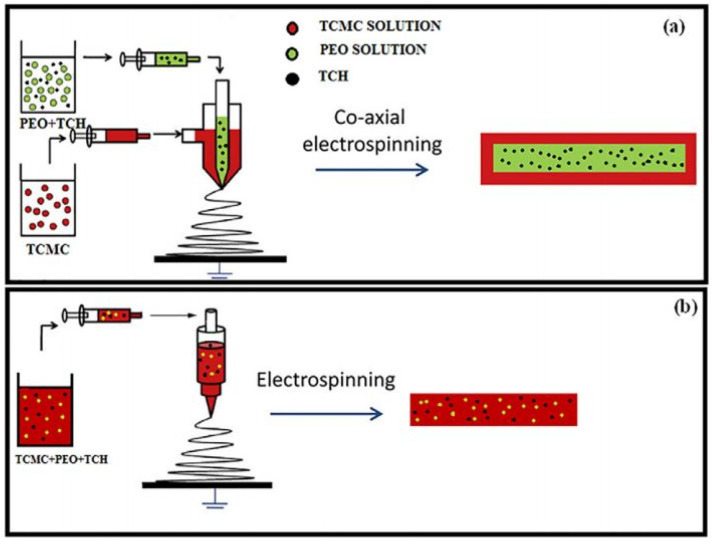
Graphical representation of the preparation of nanofibers and drug tetracycline hydrochloride (TCH) using (**a**) coaxial electrospinning and (**b**) blend electrospinning (reprinted from *Materials Science and Engineering C*, **2017**, *77*, 1117–1127, Esmaeili, A.; Haseli, M. Electrospinning of thermoplastic carboxymethyl cellulose/poly(ethylene oxide) nanofibers for use in drug-release systems, with permission from Elsevier).

In a work presented by Esmaeili and Haseli [175], a drug host based on carboxymethyl cellulose was prepared. First, the CMC was modified with methyl acrylate by grafting and then electrospun after mixing with PEO or PEG, or coaxially electrospun with one of these polymers—see Figure 21. For this work, tetracycline hydrochloride (TCH) was used as a model drug, leading to nanofibers that can be used for drug delivery and tissue engineering.

In this work, a comparison, in terms of drug release ability, between the core-shell fibers and fibers obtained from the polymer blend was made. A longer sustained release was observed over a 72 h period with core-shell fibers; however, both types of fibers exhibited excellent antimicrobial activity against Gram-positive and -negative bacteria.

## 5. Conclusions

Cellulose and cellulose derivatives in the form of nanofibers can have many applications such as in food packaging, drug delivery, biomedical materials, sensors, and filtration membranes, among others. Although there are other polymers that are more easily processed in the form of nanofibers, cellulose can be considered special for that purpose because it is a natural, biodegradable, biocompatible, and renewable polymer. Having in mind the much-needed sustainability, cellulose is a perfect candidate for use in material manufacturing. Electrospinning is currently a superior technique for cellulose-based nanofiber processing, which includes a great variety of cellulose sources, many solvents already utilized in this technique, and high-quality fibers, with many applications. On the other hand, very low production rates during ES and the use of a high-voltage electric field drives the research further to address these issues. Solution blow spinning, as an alternative to electrospinning and needleless ES, has the potential to bring the necessary qualities of the production technique to a level appropriate for industrial use. Therefore, research should be directed in exploring these techniques for cellulose-based nanofibers preparation. Additionally, it was shown that very interesting and novel IPC spinning has promising potential for cellulose- and polysaccharide-based fibrous materials, especially for biomedical applications.

In terms of the cellulose source, it was shown that there is very high potential is the utilization of cellulose ethers in the preparation of drug loading systems for controlled release. The utilization of these derivatives is driven by the fact that they are biodegradable, biocompatible, and mostly water-soluble derivatives. Additionally, the use of CNCs or CNFs combined with other cellulose or cellulose derivatives can provide all-cellulose materials, an ecofriendly solution to decrease the use of synthetic polymers.

One very important aspect was highlighted in this review, the possibility to use bottom-up spinning technologies for recycling cellulosic waste into new value-added products. In this way, much needed sustainability could be achieved.

## Figures and Tables

**Table 1:** Important physico-chemical properties of cellulose depending on the source.

Source of Cellulose	Content of Cellulose, %	Degree of Polymerization	Crystallinity, %	Degradation Temperature, °C	Water Vapor sorption, %	Water Retention, %	References
native cotton	88–96	10,000–12,000	60–90	225–300	6–7	50	[1,17,32]
various pulps	40–50	500–2000	50–56	220–330	~8	60–135	
regenerated cellulose (viscose rayon) fibers	100	250–500	25–40	~240	~12	85–95	

**Table 2:** Overview of the most utilized spinning techniques for cellulose nanofiber preparation, most common precursors and solvents used, and application of produced fibers.

Spinning Technique	Spinning Precursor	Solvents	Application	References
electrospinning	softwood and hardwood pulp, cotton, CNCs	NMMO, LiCl/DMAc, TFA, DMF, dichloroethane	biomedical application, wound dressing, drug delivery, UV protection, scaffolds	[24,38,39,40]
dry spinning	water suspension of CNFs isolated from various sources	water	high mechanical strength filaments for reinforcements in various composite materials	[41,42,43,44,45,46]
solution blow spinning	pulp	1-ethyl-3-methylimidazolium acetate, 1-ethyl-3-methylimidazolium diethyl phosphate, LiCl/DMAc	high-strength nonwovens	[47,48,49]
wet spinning	cotton pulp, CNFs	NaOH/LiOH/Urea/H_2_O, aqueous dispersions	high-strength cellulose filaments for textile materials production	[50,51,52]
dry-jet wet spinning	pulp, CNFs	1-allyl-3-methylimidazolium chloride	high-strength and thermally stable fibers	[53,54,55,56]
IPC	CNFs and TOCN	/	biological application, supercapacitors	[57,58,59]

**Table 3:** Frequently used techniques for cellulose acetate nanofibers preparation, average diameter of nanofibers, and advantages/disadvantages of each technique from the aspect of using them for cellulose acetate processing.

Spinning Technique	Nanofibers Diameter	Advantage	Disadvantage	References
electrospinning	60–5000 nm	established protocols and stable reproducible production of membranes	use of high-voltage electrical field; low production rate	[76,77]
solution blow spinning	140–1000 nm	high production rate;	obtaining of smooth fibers only with assisted spinning with other polymers	[78]
rotary jet spinning	~300 nm	/	/	[79]
electrostatic induction assisted solution blow spinning	150–1000 nm	stable preparation of nanofibers	low production rate	[80]
wet spinning	20 μm	possibility to spin cellulose acetate with CNFs or CNCs; obtaining of mechanically strong filaments	use of coagulation bath; increased use of chemicals	[81,82]

**Table 4:** Summary of various solvent systems and CA concentrations used for electrospinning of nanofibers.

Concentration of CA in Solution	Solvent System	Avg. Diameters of Nanofibers, nm	Reference
10–20%	Acetone/DMAc	100–1000	[22,85,86,87,88,89,90,91,92,93,94,95,96,97,98,99,100,101]
16%	Acetone/DMF/Trifluoroethylene	200–1000	[102]
16–17%	Acetic acid/H_2_O (75/25)	180	[103,104,105]
20%	THF/DMSO	~1000 nm	[106,107]
20%	Acetic acid (85/15)	~265	[108]
17%	Acetone/H_2_O (8:1)	400–2000	[109]
8–24%	Acetone, benzyl alcohol, methyl ethyl ketone, 1,2 Propanediol, Ethyl alcohol, DMSO	90–5000	[110]
20%	Acetone/DMF/Ethanol	150–1000	[76]
20%	DMF	~470	[111]
15%	Acetone	550	[112]
9%	Chloroform/methanol + 1% of IL 1-Butyl-3-methylimidazo-liumhexaflfluorophosphate (BMIPF_6_)	100–400	[113]
11%	Acetone/Dichlormethan/DMF	~350	[114]
10–14%	Acetic acid/acetone, Acetone/Dichlormethane, Acetic acid/H_2_O	200–1056	[115]
12%	DCM/Methanol (4:1)	720	[116]
15%	Acetone/DMF (2:1)	~150	[117]
10%	Acetic acid/H_2_O, Acetone/DMF, Acetone/DMAc	67–1500	[118]
13%	Acetone/DMAc/Methanol (2:1:2)	520–1010	[119]
20%	Acetone/DMF (6:4)	~400	[120]
16%	Acetone/DMSO	273–760	[121]
4–11%	Acetone/H_2_O (8:2)	~1100	[84]
11%	Ethanol/Acetone/DMAc (1:4:1)	/	[122]
15%	Acetone/DMAc + LiCl	/	[123]

**Table 5:** Electrospun cellulose acetate with various additives for special applications.

Substance	Loading	Application Proposed by Authors	Reference
Hydroxyapatite	Pre spinning	tissue engineering/scaffolds	[125]
AgNP	post spinning		
TiO_2_ or silicate rectorite	pre spinning	heavy metal ions removal (Pb^2+^, Cu^2+^ Cd^2+^)	[126]
Fe(CO_2_CH_3_)_2_	pre spinning	bone tissue engineering	[127]
Chitin nanowiskers	pre spinning	wound dressing, hygienic products, tissue engineering	[128]
Chitosan nanowiskers	post spinning		
FeNP	pre spinning	sensor for detection of Hg^2+^ and Pb^2+^	[129]
Carbon dots (CDs)	post spinning		
Ultrahigh silica zeolites	pre spinning	adsorption of volatile organic compounds	[130]
cationic cetylpiridinium bromide	pre spinning	air filter	[131]
SiNP and fluoroalkylsilane	post spinning	omniphobic membrane	[132]
TiO_2_ nanofibers and graphene oxide	pre spinning	antibacterial (biomedical application)	[133]
Bentonite clay	pre spinning	increased absorptive properties and cation exchange capacity	[134]
Rosmarinic acid	pre spinning	transdermal drug delivery system	[135]
poly N isopropylacrylamide	pre spinning and core shell	moisture harvesting	[136]
H_4_SiW_12_O_40_	pre spinning	photocatalytic degradation of organic compounds and dyes	[137]
Poly(glycidyl metacrilate) and Poly(acrylic acid)	post spinning	removal of heavy metal ions (Cd^2+^)	[90]
Fe_3_O_4_NP	pre spinning	removal of heavy metal ions (Pb^2+^)	[117]
Magnetite ZnO	post spinning	phenol removal	[118]
Poly(lactic acid) and polycaprolactone	pre spinning (ternary blend)	skin tissue scaffold	[138]
polyethylenimine and graphene oxide	post spinning	sensor for ammonia	[139]
perfluoro alkoxysilanes	pre spinning	oil/water separation	[124]
AgNP	pre spinning	dyes absorption from H_2_O	[140]

**Table 6:** Electrospinning of cellulosic ethers and areas of application of nanofibrous membranes.

Cellulose Ether	Degree of Substitution	Solvent Used for Electrospinning	Resulting Diameter of Nanofibers, nm	Application	References
ethylcellulose/pullulan	2.4–2.5	formic acid	167–300	active food packaging or encapsulation material	[150]
ethylcellulose/pullulan/cinammaldehyde	2.4–2.5	formic acid	200–230	antimicrobial film	[149]
elhylcellulose/gelatin	not reported	wather/ethanol/acetic acid	400–650	bioactive encapsulation, food packaging films	[151]
ethylcellulose/streptomycin	not reported	THF/DMAc	/	drug release fibers	[153]
methylcellulose	1.9	water/ethanol	50–80	enzyme immobilization	[164]
carboxymethyl cellulose/CuNP or ZnNP	not reported	LiCl/DMAc	150–200	antibacterial bandages	[169]
carboxymethylcellulose/PEO/hydroxyapatite	0.7	water	200–800	tissue scaffold	[165]
hydroxyethylcellulose	not reported	water, water with salts, surfactants or organic solvents	30–100	wound dressing, tissue scaffold	[162]

## Data Availability

Not applicable. All data are available in the review and references therein.

## Abbreviations

AGU	anhydroglucose unit
CA	cellulose (di)acetate
CNCs	cellulose nanocrystals
CMC	carboxymethyl cellulose
CNFs	cellulose nanofibrils
CTA	cellulose triacetate
DCM	dichloromethane
DMAc	*N*,*N*-dimethylacetamide
DMSO	dimethyl sulfoxide
DMF	*N*,*N*-dimethylformamide
DS	degree of substitution
EC	ethyl cellulose
ES	electrospinning
HEC	hydroxyethyl cellulose
HPC	hydroxypropyl cellulose
IL	ionic liquids
IMWS	ion mediated wet spinning
IPC	Interfacial polyelectrolyte complex spinning
KET	ketoprofen
MC	methyl cellulose
MCC	microcrystalline cellulose
Mn	number average molecular weight
NMMO	*N*-methylmorpholine-*N*-oxide
PAN	polyacrylonitrile
PVA	poly(vinyl alcohol)
PVP	polyvinylpyrrolidone
SBS	solution blow spinning
SEM	scannning electron microscopy
TBFA	tetrabutylammoniumfluoride tryhidrate
TEM	transmission electron microscopy
TEMPO	(2,2,6,6-Tetramethylpiperidin-1-yl)oxyl
TFA	trifluoroacetic acid
THF	tetrahydrofuran
TOCN	TEMPO-oxidized cellulose nanofibrils
